# Bayesian models of human navigation behaviour in an augmented reality audiomaze

**DOI:** 10.1111/ejn.15061

**Published:** 2020-12-18

**Authors:** Yumi Shikauchi, Makoto Miyakoshi, Scott Makeig, John R. Iversen

**Affiliations:** ^1^ JSPS Research Fellow Tokyo Japan; ^2^ Rhythm‐based Brain Information Processing Unit CBS‐TOYOTA Collaboration Center RIKEN Center for Brain Science Saitama Japan; ^3^ Swartz Center for Neural Computation Institute for Neural Computation University of California San Diego San Diego CA USA

**Keywords:** allocentric navigator, egocentric navigator, map generation, real‐space navigation

## Abstract

We investigated Bayesian modelling of human whole‐body motion capture data recorded during an exploratory real‐space navigation task in an “A*udiomaze*” environment (see the companion paper by Miyakoshi et al. in the same volume) to study the effect of map learning on navigation behaviour. There were three models, a feedback‐only model (no map learning), a map resetting model (single‐trial limited map learning), and a map updating model (map learning accumulated across three trials). The estimated behavioural variables included step sizes and turning angles. Results showed that the estimated step sizes were constantly more accurate using the map learning models than the feedback‐only model. The same effect was confirmed for turning angle estimates, but only for data from the third trial. We interpreted these results as Bayesian evidence of human map learning on navigation behaviour. Furthermore, separating the participants into groups of egocentric and allocentric navigators revealed an advantage for the map updating model in estimating step sizes, but only for the allocentric navigators. This interaction indicated that the allocentric navigators may take more advantage of map learning than do egocentric navigators. We discuss relationships of these results to simultaneous localization and mapping (SLAM) problem.

## Graphical abstract

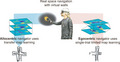

**Abbreviations**
AR: augmented reality
EEG: electroencephalography
FB: feedback‐only model
L: log‐likelihood
MBr: map‐based model with map resetting
MBu: map‐based model with map updating
RFPT: reference frame proclivity test
SLAM: simultaneous localization and mapping

## 1 INTRODUCTION

Neuroimaging studies on human navigation have been mostly conducted in stationary, virtual environments (Adomi et al., 2010; Grön et al., 2000; Janzen & van Turennout, 2004; Shikauchi & Ishii, 2016) because of limitations in neuroimaging techniques. In our companion paper in the same volume (Miyakoshi et al., “Audiomaze: EEG study on human spatial navigation using sparse augmented reality”), we demonstrated the possibility of studying sub‐second human brain dynamics during walking through actual mazes. This paradigm allowed participants to demonstrate more realistic human navigation behaviour using vestibular and kinesthetic information. Previous studies have shown that motion‐based systems contribute to navigation, and moreover lack of idiothetic information leads to differences in brain states during navigation (Park et al., 2018; Taube et al., 2013). The purpose of the sparse‐AR is to control the rate of perceptual information inflow to be greatly reduced and quantized so that the participant's map‐learning process using feedback cues from real‐world exploration can be traced down as cumulative discrete events, which allowed application of established approaches in the field of cognitive neuroscience, such as event‐related potential analysis. The same attempt was made in an international collaboration project in Berlin in which freely moving participants walked through mazes within a visually sparse‐augmented reality (AR) environment (Gehrke et al., 2018) in contrast with the auditory sparse‐AR environment of the UCSD Audiomaze paradigm in which auditory feedback is used as an alternative to wall touch feedback as experienced during real‐world navigation of physical mazes in the dark.

In order to exploit the obtained neural data, we need to have reasonable cognitive models that can be tested with the empirical data. However, constructing and validating such models requires different types of scientific background, which motivated us to conduct the current study on statistical models concerning map learning via navigation behaviour as a related but independent investigation. When we begin to explore an unknown maze, we begin with no sensory information. Thus, the status of our map learning is ‘blank slate’. However, as the same maze is explored repeatedly, behavioural data shows effects of maze learning, as confirmed by the collaborative project (Gehrke et al., 2018), as well as by modulation of brain dynamics during maze exploration in the Audiomaze paradigm (Miyakoshi et al. in the same volume).

It is argued that map learning involves a transition from egocentric to allocentric mental map configuration in which retrosplenial cortex plays a key role (Chiu et al., 2012; Gramann et al., 2010; Plank et al., 2010). Other important brain structures include the parahippocampal place area (Epstein, 2008; ﻿Epstein & Kanwisher, 1998). These brain‐informed models are useful as a standard consensus for validation and interpretation based on accumulating empirical evidence. Additionally, computational models have explored how a network of grid/place cells learns mental maps (Banino et al., 2018; Bush et al., 2015; Kubie & Fenton, 2012), see for (Epstein et al., 2017 review). However, those bottom‐up approaches alone cannot answer some of questions about the cognitive process of mental map leaning. For example, we may ask the following question—what happens if someone does not shift to an allocentric mental mapping strategy during maze learning and stayed in egocentric mental mapping strategy? How much is the difference in efficiency reflected in the behavioural data? To quantify the effect of the model differences, we need different principles and approaches.

In the current study, we asked the effect of map learning on navigation behaviour within a basic Bayesian framework to test the advantage of map learning model for estimating actual maze exploration behaviour. The process of map learning in an unknown environment has been studied in the field of robotics rather than in neuroscience (Bailey & Durrant‐Whyte, 2006; Durrant‐Whyte & Bailey, 2006). It is a complicated problem that requires the identification of the current position and updating the map at the same time. This chicken‐or‐egg problem is called the SLAM (simultaneous localization and mapping) problem. However, SLAM can be considered a solved problem theoretically and various implementations have been proposed. Inspired by those efforts, we prepared three learning models to study the differences: no learning, single‐trial limited map learning, transferred learning across repeated trials. We required our participants to solve the maze of the same shape three times, without giving any prior information about the shape. Then, the following hypothesis can be considered: egocentric navigators use potentially different spatial knowledge between trials, thus the maps learnt in the past trials cannot be fully utilized (single‐trial limited map learning). Since allocentric navigators use a common spatial knowledge system, what has been learned in the past trials can be utilized in the second and third trials (transferred learning).

The validity of each of these models should be determined by the motion capture data recorded from a group of participants in the Audiomaze project. In addition, one of the key questions in the Audiomaze paradigm, namely characterization of the egocentric versus allocentric navigational styles, should be tested by splitting the participants into two subgroups based on their responses to an online navigation style survey.

## 2 MATERIALS AND METHODS

### 2.1 Participants

Participants were 16 healthy adults (9 female, age *M* = 26.5, *SD* = 6.2, range 20–41). They were recruited from the student community of the University of California San Diego. After receiving verbal and written explanations of study requirements, and prior to any study procedures, all parents/participants provided written informed consent/assent as approved by the UCSD Human Research Protection Program.

### 2.2 Navigational style screening

Individuals differ in their spatial abilities and the strategies underlying spatial navigation (Gramann, 2013; Wolbers & Hegarty, 2010). Such individual differences may play a role in behaviour and brain responses (Gramann et al., 2010; Lin et al., 2015). Participants were pre‐tested and categorized with respect to their preferred use of an egocentric or an allocentric reference frame during navigation using the online reference frame proclivity test (RFPT; https://www.silc.northwestern.edu/spatial‐reference‐frame‐proclivity‐test/; Goeke et al., 2015; Gramann et al., 2005). These participants were separated into subgroups of eight egocentric and eight allocentric navigators.

### 2.3 Motion capture data recording

**FIGURE 1:**
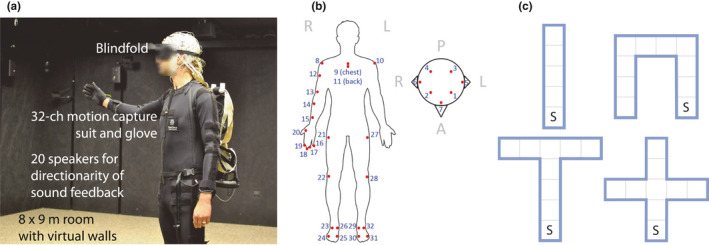
(a) Experimental environment and set up for participants. The experimental room had 8 × 9 meter size, equipped with 12 + 8 speakers (on walls and ceilings, respectively), 20 motion capture cameras, and sound‐absorbing wall materials painted in black. (b) Configuration of the motional capture markers. Participants were in the special suit that had 32 LED markers on the head, torso, right arm including hand, and both legs. (c) The shapes of the mazes. Each shape of the maze was repeated three times in a row

An example scene from the experiment is shown in Figure 1 (the room light was left on to take this picture; during the task, all lights were turned off). Participants wore a motion capture suit (PhaseSpace Inc.) with a custom 32‐marker configuration to record motion data with a sampling rate of 480 Hz. A LED marker driver including a battery was stored in a backpack. There were 12 cameras mounted on the walls and eight cameras on the ceiling to detect positions of the markers and generate xyz room‐coordinate values for marker. The participants performed an exploration of the space in front of them by reaching out with their dominant arm and hand to test for the presence of a wall via the auditory “wall touch” feedback (below, “hand beep”). In addition, virtual “wall touches” with their head produced another feedback sound (“head beep”). Each participant underwent three consecutive trials exploring each of four differently shaped mazes, giving 12 trials total per participants. Due to technical errors, data from seven trials were not usable. The final number of the trials analysed was 185.

### 2.4 Data preprocessing

**FIGURE 2:**
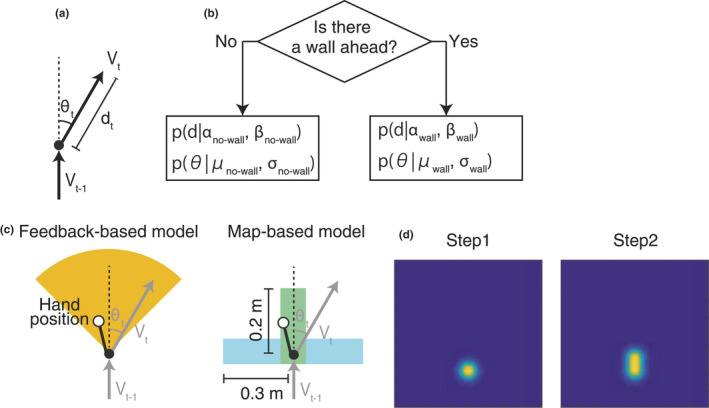
Schematic representation of the feedback‐based model and map‐based models. (a) The motion vector V was defined as the change of the torso position every 500 ms, specified by angle and distance. (b) All models switch between two step/angle distrubutions based on a prediction of whether there is a wall ahead or not. (c) The feedback model simply predicts a wall ahead when a wall is detected by hand/head sensors (left). The yellow area shows the angular range used for the wall detection. The map‐based models (right) detect a wall ahead based on a mental wall‐probability map learned from previous navigation in the maze. The definition of wall ahead was that there is high probability of a wall in the green area ahead but not in the light blue areas to the sides. (d) The mental map consists of wall probability and no‐wall probability maps updated each step based on the presence or absence of sensory feedback. Here, the nowall probability is updated after a forward step

After calculating a moving average over five timepoints, the motion capture data were downsampled to 2 Hz to obtain marker locations and an individual's motion vector $V_{t}^{*}$ using position changes in their centre of mass computed from the set of motion capture markers on their torso (Figure 2a).

### 2.5 Modelling of navigation behaviour

We proposed three computational models of participants' movements (characterized by step size *d* and turn size θ at every 500 ms sample interval): a *feedback‐only model* and two map‐based models, *a map resetting model* and *a map updating model*. The first modelled participant steps and turns from current auditory feedback alone, whereas the latter two models generated a cognitive map using accumulated past observations and used that to guide future actions. The map resetting model generated a new mental map for each trial, whereas the map updating model inherited the mental map generated in the past trials.

Formally, each model assumed that the probability of step size at time *t*, which is non‐negative and continuous, is modelled as a Gamma distribution.

$$p\left(d\right)=\frac{1}{\Gamma \left(\alpha \right)\beta^{\alpha }}d^{\alpha ‐1}e^{‐d/\beta }.$$

Here *α* and *β* are a shape parameter and a scale parameter respectively. Г(*α*) is the Gamma function, an exponential family of continuous probability distributions supported on the semi‐infinite interval [0, ∞). The probability of rotating at time *t* by an angle θ was modelled as a von Mises distribution

$$p\left(\theta \right)=\frac{\exp\left\{\left(\theta ‐\mu \right)\right\}}{2\pi I_{0}\left(\sigma \right)}.$$

Here *I*_0_(*σ*) is the modified Bessel function of order 0, and the parameters *μ* and *σ* are analogous to the mean and variance in the normal distribution.

We assumed that the motion changes depending on whether the space ahead of the next forward step is estimated by the subject to wall‐touch or not. (Figure 2b). Each model estimated the wall‐touch status (wall or no‐wall) based on different criteria (see Section 2.7, below). Thus, each model had eight free parameters: α_no‐wall_, β_no‐wall_, α_wall_, β_wall_, μ_no‐wall_, σ_no‐wall_, μ_wall_ and σ_wall_. We modelled the fact that participants needed to estimate their current positions based only on their recollected movement history, as they could not see while exploring. Thus, our model has an estimated motion vector, *v_t_* instead of the measured motion *V* ^*^ as follows:

$$V_{t}=V_{t}^{*}+\left|V_{t}^{*}\right|\varepsilon .$$

Here *ε* is Gaussian noise, *N*(0,(0.002 0.0005 0.0005 0.002)). From Gaussian noise accumulation, the deviance in this model of their estimated position ${\left(X,Y\right)}_{\text{body}}$ from their measured position ${\left(X^{*},Y^{*}\right)}_{\text{body}}$ behaves as a random walk process as follows:

$${\left(X_{t},Y_{t}\right)}_{\text{body}}={\left(X_{1}^{*},Y_{1}^{*}\right)}_{\text{body}}+\sum_{i=1}^{t}V_{i}.$$

Additionally, we described the estimated right hand position ${\left(X,Y\right)}_{\text{hand}}$ as follows:

$${\left(X_{t},Y_{t}\right)}_{\text{hand}}=U_{t}^{*}+{\left(X_{1}^{*},Y_{1}^{*}\right)}_{\text{body}}+\sum_{i=1}^{t}V_{i}$$

Here, $U_{t}^{*}$ indicates a vector between the measured hand position ${\left(X_{t}^{*},Y_{t}^{*}\right)}_{\text{hand}}$ and the measured body position ${\left(X_{t}^{*},Y_{t}^{*}\right)}_{\text{body}}$

### 2.6 Generation of the mental map

Two mental maps in the map‐based models were initialized as two‐dimensional uniform distributions corresponding to a wall probability and a no‐wall probability at each spatial location, *P*(*X*,*Y*)_wall_ and *P*(*X*,*Y*)_no‐wall_. At each timestep the observation (wall‐proximity/‐touch sound feedback → ‘wall’; no feedback → ‘no‐wall’) is represented as a two‐dimensional Gaussian distribution. When receiving hand wall feedback at ${\left(X_{t},Y_{t}\right)}_{\text{hand}}$, then a Gaussian with mean at the estimated hand position is added to the wall probability distribution, at ${\left(X_{t},Y_{t}\right)}_{\text{body}}$ a Gaussian is instead added to the no‐wall probability distribution at the position of the body centre of mass (Figure 2d). Occasional steps with head contacting the wall did not update the model. The covariance matrix of each Gaussian was (0.002 0 0 0.002) yielding a symmetric distribution with full‐with half‐maximum of ~10 cm at each touch point. The final mental maps defined were thus two two‐dimensional Gaussian mixture distributions.

### 2.7 Criteria for judging wall presence

In the feedback‐only model, the participant determined presence of a wall 'ahead' by reaching forward at an angle between θ_t−1_ – 45° and θ_t−1_ + 45° and receiving a wall proximity/touch feedback, or by receiving the “head beep” (Figure 2c). In the map‐based models, they determined presence of a wall ahead, based only on the internal probability maps, when the following conditions were satisfied: (a) the probability of a wall being 5–20 cm ahead is higher than the model's wall probability in the model's current estimated position, and the no‐wall probability of the step‐ahead position is lower than that of the current estimated position, and (b) no‐wall probability in the space within 5–30 cm to the right/left is higher than the model's no‐wall probability in the estimated current position, and the wall probability of the lateral space is lower than that of the current estimated position. This probability is maximal in the condition when the participant is facing a wall, with the corridor stretching left and right.

### 2.8 Evaluation of the model fit

We evaluated validity of the use of the mental maps during spatial navigation by quantifying participant's behaviour across the 3 maze trials. We calculated the step size *d* and turning angle θ from the motion vector V* and the determination of whether there was a wall ahead (Figure 2 and above). Then, we estimated model parameters of the Gamma distribution of step size by maximum likelihood estimation for the wall steps and the no‐wall steps respectively. The model parameters *μ*_•_ and *σ*_•_ were estimated in the same way. Using the model parameters estimated for each model, the log‐likelihood of step size *d* and turning angle θ were obtained.

## 3 RESULTS

### 3.1 Map‐based Models fit human step size

The estimated model parameters are listed in Table 1. Expected values of step size $\left(E\;\left(X\right)=\alpha_{\cdot }\beta_{\cdot }\right)$ were larger in the no‐wall steps than in the wall steps (no‐wall (mean [cm] ± *SEM*) vs. wall, feedback‐only model 6.46 ± 0.27 vs. 2.43 ± 0.05; map‐reset model 6.76 ± 0.23 vs. 2.13 ± 0.02; map‐update model 6.86 ± 0.24 vs. 2.12 ± 0.02). Similarly, the estimated mean of turning angle (*μ*_._) was larger in the no‐wall steps than in the wall steps. This is counterintuitive since wall detection in front would lead to a turn. It may be considered that this is because the step sizes become large in no‐wall steps, and the turning angles become large unintentionally in the absence of visual feedback. Normalized with the expected values of the step size, it becomes as follows: (mean [°] ± *SEM*) feedback‐only model 0.22 ± 0.01 vs. 0.64 ± 0.026; map‐reset model 0.31 ± 0.01 vs. 0.80 ± 0.02; map‐update model 0.35 ± 0.01 vs. 0.79 ± 0.02). Our proposed models were consistent with reasonable navigation behaviour, turning in wall steps, and moving forward in no‐wall steps.

**TABLE 1:** Estimated parameters

Model	α_no‐wall_	β_no‐wall_	α_wall_	β_wall_	μ_no‐wall_	σ_no‐wall_	μ_wall_	σ_wall_
FB	2.48 ± 0.46	4.72 ± 0.26	1.41 ± 0.02	1.75 ± 0.03	1.15 ± 0.04	9.46 ± 0.29	1.10 ± 0.01	2.01 ± 0.03
MBr	1.12 ± 0.03	6.37 ± 0.19	1.18 ± 0.01	1.86 ± 0.03	1.84 ± 0.08	9.83 ± 0.39	1.02 ± 0.01	2.26 ± 0.04
MBu	1.10 ± 0.03	6.56 ± 0.20	1.17 ± 0.01	1.85 ± 0.02	2.27 ± 0.11	8.99 ± 0.39	1.01 ± 0.01	2.39 ± 0.07

Note

Mean ± *SEM*.

Abbreviations: FB, feedback‐based model; MBr, map reset model; MBu, map update model.

**FIGURE 3:**
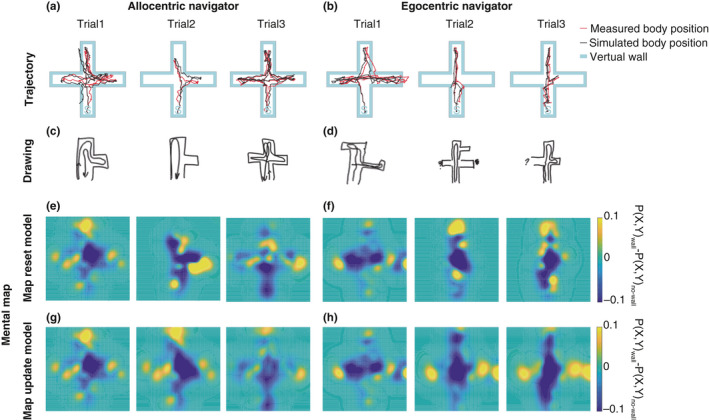
Movement trajectories and estimated mental models for two participants, an allocentric‐ (left) and egocentric‐ (right) style navigator. (a, b) Red and black traces indicate the measured and estimated trajectories, respectively. From left to right, Trial 1, 2, and 3. (c, d) Participants wrote down the maze shape they estimated and the trajectory they moved through it after each trial. (e, f) Estimated mental maps by the map reset model. (g, h) Estimated mental maps by the map update model. From left to right, Trial 1, 2 and 3. The wall probability was subtracted from the no‐wall probability for visualization, with yellow showing 'wall' probability and blue the 'no‐wall' probability

**FIGURE 4:**
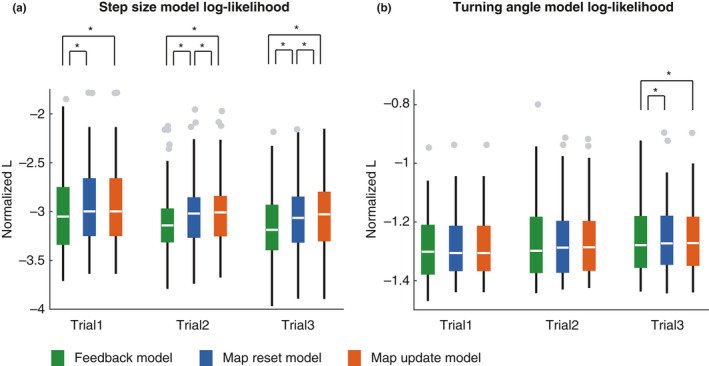
Prediction performance of estimated step sizes (a) and turning angles (b). The log‐likelihood (L) depends on the number of steps and so was normalized by dividing by the number of steps taken during the current exploration to correct the imbalance in the number of steps between participants and trials. There are significant differences between models' ability to predict step size in all trials and turning angle in Trial 3 (Friedman's test, *p* < .01, *df* = 2). All asterisks in (a) and (b) indicate significant differences between the two map‐based models and the feedback‐based model (post hoc two‐sided Wilcoxon signed rank test, *p* < .001, Bonferroni corrected for multiple comparisons). The bottom and top edges of the box indicate the 25th and 75th percentiles, respectively. Gray dots indicate outliers which are greater than q3 + 1.5 × (q3 − q1) or less than q1 − 1.5 × (q3 − q1), where q1 and q3 are the 25th and 75th percentiles, respectively. A whisker extends to the most extreme data value that is not an outlier

Figure 3 shows examples of movement trajectories and mental maps generated by map‐based models. We compared model log‐likelihood across the feedback‐only, map resetting, and map updating models. First, we tested step sizes. There were significant differences across these three models in all three trials (Friedman test, *p* < .0001). Post‐hoc tests revealed significantly better step size estimation in the map‐based models than the feedback‐only model (Wilcoxon signed‐rank test, *p* < .0001, Bonferroni‐corrected for multiple comparisons; Figure 4a). Secondly, we tested turning angle. We found significant difference only in trial 3 (Friedman test, *p* < .0001; Wilcoxon signed‐rank test, *p* < .0001, Bonferroni‐corrected for multiple comparisons; Figure 4b). These results suggest that the using a mental map increased the prediction accuracy of navigation behaviour, especially moving distance, than the feedback‐only model.

In addition, the performance in step‐size prediction was significantly higher in the map update model than that of the map resetting model when considering only trials 2 and 3, (the two models are the same, by definition, in Trial 1; post‐hoc Wilcoxon signed‐rank test, *p* < .001, Bonferroni‐corrected for multiple comparisons). The results suggest that mental maps not only play a role of working memory such as holding the route that has been taken so far, but also contributes to detect the map geometry and make movement more efficient.

### 3.2 Mental map prevents wall oversight

**FIGURE 5:**
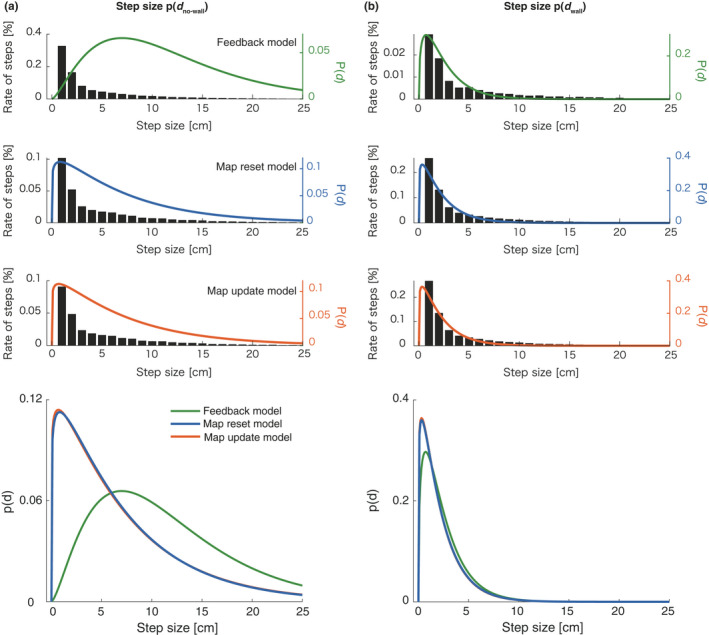
Comparison between estimated probability of step size *p*(*d*) and measured step size. Each step was divided into no‐wall condition (left) and wall condition (right) based on the wall detection criterion of each model. Black bars indicate rate of the number of steps with each step size across all participants and all trials. Green, blue and red lines show probability distribution function of estimated Gamma distributions for the three models. In the lowest panels, the estimated probabilities drawn separately in the upper panels are overwritten for comparison

To describe in detail how the results for the map‐based models differed from those for the feedback‐only model, we separated the step sizes into wall detected steps and no‐wall detected steps. We found that the feedback‐only model detected a wall ahead in 9.3% steps, while the map‐based models detected a wall ahead in 68.6% and 71.1% steps, respectively (Figure 5). It was confirmed that the map‐based models efficiently switched between the two strategies.

### 3.3 Allocentric navigators add up maps

**FIGURE 6:**
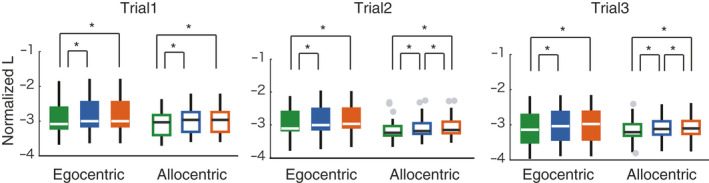
Differences in step‐size prediction between egocentric participants (filled boxes) and allocentric participants (open boxes). From left to right, Trial 1, 2 and 3. There are significant differences between models in all panels (Friedman test, *p* < .0001). Asterisks: significant differences (post hoc two‐sided Wilcoxon signed rank test, Bonferroni correction, *p* < .05)

As shown in Figure 3, there were individual differences in the ability to learn the maze geometry. We asked a question whether our models can help understand these individual differences. We compared model probabilities between egocentric navigators (*n* = 8) and allocentric navigators (*n* = 8), as determined by a pre‐experiment test of each navigator's proclivity (RFPT). We found that allocentric navigators showed significant differences between the map resetting model and the map updating model, while egocentric navigators did not show any significant difference between them (Figure 6). This result suggested that allocentric navigators' behaviour was better fit by a model that learns the map across trials.

## 4 DISCUSSION

In this study, we built three Bayesian models representing a feedback‐only model (no map learning), a map resetting model (map learning limited to within a single trial), and a map updating model (transferring learning across repeated trials). Our primary goal was to determine which of the three models explains the human real‐space navigation data best. The results showed that compared with the feedback‐only model, both map learning models showed better step size estimation. Moreover in Trials 2 and 3, a carry‐over effect in map updating took effect such that the step size estimation was better for the map updating model than the map resetting model. Also for turning angle estimation, both map‐based models showed an advantage in prediction over the feedback‐only model. These results are consistent with our understanding that human navigators do develop map learning even in a highly controlled and artificial environment such as the Audiomaze (Miyakoshi, et al, this issue). The results are in line with empirical data recorded in a similar environment using sparse‐AR visuospatial feedback in an experiment in which participants spent less time and moved faster in the maze as trials were repeated (Gehrke et al., 2018).

We next asked if individual differences in navigation style might affected model performance. Splitting the participants into two subgroups, one for allocentric navigators and the other for egocentric navigators, showed a similar advantage of the map‐based models over the feedback‐only model in both groups. In the allocentric navigators only, there was a further advantage of the map updating model over the map resetting model. The observation that the map learning (map‐updating) model best fit the allocentric navigator behavior is consistent with the idea that allocentric navigators may have been better in exploring the maze based on the mental maps they built, and this advantage may be further reinforced by repeating the navigation.

As far as we know, this is the first evidence that individual differences in human navigational style can be explained in terms of Bayesian modelling of map learning. The result will provide additional information that can be used in interpreting electroencephalography (EEG) and behavioural differences between the egocentric versus allocentric navigators (Miyakoshi, et al, this issue), in particular the finding that allocentric navigators may take advantage of learning better than egocentric navigators.

To estimate one's current position in the dark and to learn a map in an unknown environment is difficult. Estimation of current position requires map information, while generating a map requires current position information. SLAM is a solved problem in the robotics community and has also been implemented in various kinds of products such as a robotic cleaner (Durrant‐Whyte & Bailey, 2006). SLAM algorithms including neural networks of grid cells and head direction cells have already been proposed in several studies (Zeng & Si, 2017; Zeng et al., 2020). These models successfully built coherent maps in robot navigation or car navigation with a monocular camera.

The study of spatial memory system of mammalian brains is one of the most advanced fields in neuroscience. Although it is well known that there are place cells that respond to a specific position like GPS and grid cells that provide metric system, it is unclear how the SLAM problem is solved in the brain. In this study, we demonstrated computational modelling of human navigation behaviour with mental mapping inspired by SLAM research in robotics. As a result, map learning seems help human navigation, especially in later trials with accumulated knowledge of the environment and in an allocentric navigator. The results could be useful in assessing and facilitating individual navigation abilities.

We demonstrated that map learning using proprioceptive and auditory feedback, not proprioceptive, visual and haptic feedback as heavily used in everyday map learning. Research on blind people navigating an unknown environment indicates that haptic and auditory feedback supplies information that helps in map learning of new spaces (Lahav & Mioduser, 2003). Our results are consistent with the idea that information integration across modalities is key for navigation.

Using our mobile brain/behavioural imaging (MoBI) setup, we measured navigation behaviour with motion‐based systems such as vestibular sensation. All our models estimated the motion (*V_t_*) as the measured motion ( $V_{t}^{*}$) plus the Gaussian noise (*ε*) proportional to the distance ( $\left|V_{t}^{*}\right|$). Since there is no visual feedback, it is a realistic assumption that the estimation of the current position deviated from the actual position as the steps progress. Further studies are needed in order to estimate a truer mental map by building an advanced model that can express individual differences (highly/less accurate person) and situation dependence (wall/no‐wall steps) of position estimation with motion‐based systems.

In conclusion, we asked the effect of map learning on human navigation behaviour in a basic Bayesian framework so that the advantage of map learning could be tested. We measured human navigation behaviours in an auditory sparse‐AR environment in the Audiomaze paradigm. Our participants explored real‐sized mazes based on auditory feedback plus vestibular and kinesthetic information. While they explored each maze shape three times, their step sizes were estimated constantly better by the map‐based learning models than the feedback‐only model. We interpreted these results as Bayesian evidence of human map learning on navigation behaviour. Moreover our model‐based analysis suggested that while allocentric navigators update maps across trials (transfer learning), egocentric navigators may not be able to reuse maps efficiently (single‐trial‐limited map learning). This interaction indicated that the allocentric navigators may take more advantage of map learning than do egocentric navigators. Our map learning models allow visualization of the user's development of a mental map step by step. These appear to be a promising tool for EEG analysis of data collected during navigation in the real world using continuous data without clear event onsets.

## CONFLICT OF INTEREST

The authors declare no competing financial interests.

## AUTHOR CONTRIBUTIONS

Y.S., M.M. and J.R.I. contributed to model conceptualization and investigation. S.M., J.R.I. and M.M. contributed to experiment conceptualization. Y.S. contributed to methodology and formal analysis. M.M. and Y.S. contributed to writing—original draft. M.M., J.R.I. and S.M. contributed to writing—review and editing. Y.S and M.M. contributed to visualization. S.M., J.R.I and Y.S contributed to funding acquisition.

### PEER REVIEW

The peer review history for this article is available at https://publons.com/publon/10.1111/ejn.15061.

## Data Availability

Sample empirical data and Matlab code are available at https://sccn.ucsd.edu/wiki/Audiomaze.
